# Comparing Immune Responses to Inactivated Vaccines against SARS-CoV-2 between People Living with HIV and HIV-Negative Individuals: A Cross-Sectional Study in China

**DOI:** 10.3390/v14020277

**Published:** 2022-01-28

**Authors:** Xiaojie Huang, Ying Yan, Bin Su, Dong Xiao, Maohe Yu, Xia Jin, Junyi Duan, Xiangjun Zhang, Shimin Zheng, Yuan Fang, Tong Zhang, Weiming Tang, Lunan Wang, Zixin Wang, Junjie Xu

**Affiliations:** 1 Clinical and Research Center for Infectious Diseases, Beijing Youan Hospital, Capital Medical University, Beijing 100069, China; huangxiaojie78@ccmu.edu.cn (X.H.); binsu@ccmu.edu.cn (B.S.); youanduanyi@163.com (J.D.); zt_doc@ccmu.edu.cn (T.Z.); 2 National Center for Clinical Laboratories, Beijing Hospital, National Center of Gerontology, Institute of Geriatric Medicine, Chinese Academy of Medical Sciences, Beijing 100730, China; yyan511@nccl.org.cn; 3 Rainbow Clinic of Beijing Jingcheng Skin Hospital, Beijing 100101, China; hivct@me.com; 4 Department of AIDS/STD Control and Prevention, Tianjin Center for Disease Control and Prevention, Tianjin 300011, China; yumaohe@tj.gov.cn; 5 AIDS Healthcare Foundation (AHF), Beijing 100088, China; jinxiajiangsu@126.com; 6 Department of Public Health, The University of Tennessee, Knoxville, TN 37996, USA; xzhan140@utk.edu; 7 Department of Biostatistics and Epidemiology, East Tennessee State University, Johnson City, TN 70300, USA; zhengs@etsu.edu; 8 Department of Health and Physical Education, The Education University of Hong Kong, Hong Kong 200092, China; lunajoef@gmail.com; 9 University of North Carolina Project-China, Guangzhou 510095, China; 10 JC School of Public Health and Primary Care, Faculty of Medicine, The Chinese University of Hong Kong, Hong Kong 666888, China; 11 Clinical Research Academy, Peking University Shenzhen Hospital, Peking University, Shenzhen 518036, China

**Keywords:** people living with HIV, inactivated SARS-CoV-2 vaccines, self-reported adverse events, neutralizing activity responses against authentic SARS-CoV-2, total antibody specific to SARS-CoV-2, SARS-CoV-2 IgG antibody, antigen-specific T-cell immune response

## Abstract

This study compared the immunogenicity of inactivated SARS-CoV-2 vaccines between people living with HIV (PLWH) and HIV-negative individuals. We recruited 120 PLWH and 53 HIV-negative individuals aged 18–59 years who had received an inactivated SARS-CoV-2 vaccine in two Chinese cities between April and June 2021. Blood samples were tested for immunogenicity of the inactivated SARS-CoV-2 vaccines. The prevalence and severity of adverse events associated with SARS-CoV-2 vaccines were similar between PLWH and HIV-negative individuals. The seropositivity of neutralizing activity against authentic SARS-CoV-2, of the total amount of antibody (total antibody) and of S-IgG were 71.3%, 81.9%, and 92.6%, respectively, among fully vaccinated PLWH. Among all participants, PLWH had lower neutralizing activity, total antibody, S-IgG, and T-cell-specific immune response levels, compared to HIV-negative individuals, after controlling for types of vaccine, time interval between first and second dose, time after receiving the second dose, and sociodemographic factors. PLWH with a longer interval since HIV diagnosis, who received their second dose 15–28 days prior to study commencement, and who had an interval of ≥21 days between first and second dose had higher neutralizing activity levels. The immunogenicity of the inactivated SARS-CoV-2 vaccines was lower among PLWH as compared to HIV-negative individuals. Vaccination guideline specific for PLWH should be developed.

## 1. Introduction

Globally, about 38 million people are living with HIV [1]. Antiretroviral therapy (ART) has been shown to suppress viral replication, restore CD4+ T-cell counts, rebuild immune function, and decrease morbidity and mortality among people living with HIV (PLWH) [2,3]. However, CD4+ T-cell recovery is incomplete despite viral suppression in some PLWH [4]. There is conflicting evidence as to whether PLWH have an increased risk of SARS-CoV-2 infection [5,6]. Several systematic reviews and non-systematic reviews found comparable mortality and morbidity of SARS-CoV-2 between PLWH and HIV-negative individuals [7,8,9,10,11]. SARS-CoV-2 vaccination is important for all patients with potentially decreased immune responses (e.g., patients with autoimmune diseases or who are receiving immunosuppressive agents, and PLWH) [12]. Both international health authorities and Chinese national guidelines recommend SARS-CoV-2 vaccination for PLWH regardless of their immune status [13,14,15].

PLWH are considered a priority group for vaccination in many countries [15]. However, there are concerns that PLWH might have a suboptimal response to SARS-CoV-2 vaccination. More importantly, less than 3% of the participants in the reported SARS-CoV-2 vaccine efficacy trials are PLWH, and the data for vaccine safety and immune response are insufficient [16,17,18,19]. Some studies have compared the safety and immunogenicity of mRNA (Pfizer BNT162b2 and Moderna mRNA-1273) or adenovirus vector (Oxford/AstraZeneca AZD1222) SARS-CoV-2 vaccines between HIV-negative individuals and PLWH who have viral suppression and high CD4+ T-cell levels (median around 700) [20,21,22,23,24]. These studies showed that SARS-CoV-2 vaccines were safe for PLWH, and that there was no between-group difference in adverse events [20,21,22,23,24].

There are two inactivated SARS-CoV-2 vaccines manufactured by Chinese companies that are approved for emergency use by the World Health Organization (WHO) (Sinopharm and Sinovac CoronaVac) [25,26]. More than three billion doses of these vaccines have been supplied to more than 40 countries [27]. No study has compared PLWH and HIV-negative individuals when it comes to the immunogenicity and safety of the inactivated SARS-CoV-2 vaccines. Such evidence is important to address COVID-19 vaccine hesitancy among PLWH and to implement booster doses for this group [28]. Previous findings on mRNA/adenovirus vector vaccines might not be applicable to PLWH receiving inactivated SARS-CoV-2 vaccines [20,21,22,23,24]. Moreover, it is unclear whether PLWH with lower CD4+ T-cell counts and with detectable HIV viral loads would have similar immunogenicity as HIV-negative individuals, as these PLWH were excluded by the aforementioned studies [20,21,22,23,24]. Furthermore, given the relatively short follow-up period in previous studies, there is no consensus about the long-term immunogenicity of SARS-CoV-2 vaccines among PLWH [20,21,22,23,24].

This study aims to address these knowledge gaps by comparing the immunogenicity and adverse events associated with these vaccines between PLWH and HIV-negative individuals after vaccination. This study also investigated factors correlated with levels of neutralizing activity against authentic SARS-CoV-2, the total antibody specific to SARS-CoV-2, SARS-CoV-2 IgG antibody against the receptor-binding domain (RBD) of the spike protein (S-IgG), and antigen-specific T-cell immune response among PLWH.

## 2. Materials and Methods

### 2.1. Study Design

This cross-sectional study was conducted in two Chinese metropolitan cities (Beijing and Tianjin) between April and June 2021. Participants included PLWH and HIV-negative individuals who had received at least one dose of inactivated SARS-CoV-2 vaccine.

### 2.2. Participants

The inclusion criteria for PLWH included the following: (1) aged 18–59 years; (2) willing to participate in the study activities, including survey and blood sample collection, and relevant laboratory testing; (3) having received at least one dose of inactivated SARS-CoV-2 vaccine (Sinovac CoronaVac or Sinopharm); and (4) having received an HIV diagnosis confirmed by HIV-1/2 Western blot assay. Exclusion criteria included the following: (1) presence of severe hearing loss, impaired vision, or intellectual disability observed by the interviewers; or (2) a history of SARS-CoV-2 infection, major psychiatric illness (schizophrenia or bipolar disorder) or neurocognitive impairment based on clinician’s assessment of their medical records. HIV-negative individuals shared the first three inclusion criteria and both exclusion criteria with PLWH. HIV serostatus was confirmed by Abbott ARCHITECT HIV Ag/Ab Combo assay.

### 2.3. Recruitment and Data Collection

Recruitment for PLWH was facilitated by two community-based organizations (CBOs), one in each city. These two CBOs have provided services to PLWH and HIV high-risk populations and worked closely with HIV clinical service providers. WeChat is the most commonly used social media application for the CBOs to communicate with PLWH clients. CBO staff posted the study recruitment information in the WeChat public accounts of their organizations. Interested PLWH contacted CBO staff through private WeChat messages, phone calls, or messages via other instant messaging applications. CBO staff screened participants’ eligibility, briefed them about the study purpose and procedures, assured them that identifiable information would be kept confidential, and informed them that their refusal to participate would have no consequences. The recruitment of HIV-negative individuals was conducted in community hospitals. The hospital staff identified vaccinated individuals based their patient records and contacted them by telephone to invite them to participate.

PLWH and HIV-negative individuals interested in joining the study were invited to visit one of two clinics, based in each city. Whilst prospective participants were on site, project staff obtained their written informed consent. All participants also completed a 10-min self-administered questionnaire whilst on site.

### 2.4. Blood Sample Collection and Laboratory Procedures

After completion of the survey, trained nurses collected two lithium heparin anticoagulated vacuum blood collection tubes (BD) of whole blood (10 mL), two EDTA anticoagulated vacuum blood collection tubes (BD) of whole blood (10 mL), and one SST blood collection tube of whole blood (5 mL). One tube of lithium heparin salt anticoagulated whole blood and one tube of EDTA anticoagulated whole blood were placed at room temperature. They were assayed for T-cell-specific immune response within 8 h and for CD4+ T-cell count within 48 h, respectively. The other three tubes of whole blood were centrifuged at 1300 relative centrifugal force (RCF) for 10 min, and the upper plasma/serum layers were transferred into lyophilized tubes of no less than 1.2 mL each, then stored at −20 °C for the detection of SARS-CoV-2 combined antibody and neutralizing activity, as well as HIV viral load.

SARS-CoV-2 neutralizing activity measurement. The neutralizing activity to authentic SARS-CoV-2 (virus strain SARS-CoV-2/human/CHN/CN1/2020, GenBank number MT407649.1) was quantified using a micro cytopathogenic effect (CPE) inhibition assay with a minimum four-fold dilution, as reported before [29]. This neutralization assay is considered the gold standard measurement of neutralizing activity. The positive geometric mean titer (GMT) of the neutralizing activity to authentic SARS-CoV-2 was 8.

SARS-CoV-2 antibody combined testing. All samples were tested for SARS-CoV-2 specific total antibody and S-IgG antibodies using Chemiluminescence assay (CLIA) kits (Beijing Wantai Biological Pharmacy Enterprise Co., Ltd., Beijing, China). The total antibody was detected by double-antigen sandwich-based method, which could detect the total amount of SARS-CoV-2-specific IgG, IgM, and IgA. S-IgG was detected by indirect method. The positive cut-off for the abovementioned tests was 1.0.

T-cell-specific immune response. The T-cell-specific immune response was tested using the IFN-γ release assay (Beijing Wantai Biological Pharmacy Enterprise Co., Ltd., Beijing, China). Briefly, 1.5 mL of heparin, blood was distributed into a test tube containing specific SARS-CoV-2 S-antigen (T tube), a negative control tube (N tube), and a positive control tube (P tube) within 8 h. The tubes were inverted and mixed 5 times, then incubated in 37 °C for 20–24 h. Then, the plasma was collected after centrifuging at 3000 RCF for 10 min and assessed for IFN-γ level. If the level of T tube minus N tube resulted in a value greater than 30 pg/mL the result was considered positive.

HIV viral load. Viral load of PLWH was tested using HIV quantitative assay (Zhuhai Livzon Diagnostics Inc., Zhuhai, China). The limit of quantitation (LOQ) of this assay was 60 copies/mL.

CD4+ cell count measurement. The assay was performed using flow cytometry testing methods (BD Biosciences, San Jose, CA, USA) in accordance with the China National Guideline for Detection of HIV/AIDS (version 2020) [30].

Background characteristics of the participants. All participants reported age, gender, and presence of chronic conditions (i.e., hypertension, hyperlipidemia, cancer, chronic cardiovascular, lung, liver, or kidney diseases, and diabetes mellitus). Characteristics related to HIV infection and SARS-CoV-2 vaccination were extracted from medical records.

Adverse events related to SARS-CoV-2 vaccination. A checklist was used to assess local adverse events (pain, redness, itching, swelling, induration, and skin rashes in the arm where the shot was given) and systematic adverse events (fatigue, malaise, headache, dizziness, lethargy, joint pain or muscle ache, fever, nausea, vomiting, diarrhea, and others) within one month after receiving a SARS-CoV-2 vaccine. Participants rated the severity of the aforementioned adverse events (1 = very mild, 2 = mild, 3 = moderate, 4 = severe, and 5 = very severe).

### 2.5. Sample Size Planning

Previous studies showed that the positive rate for SARS-CoV-2 neutralizing activity was about 90% among HIV-negative individuals who received inactivated SARS-CoV-2 vaccines [29]. There were no data on seropositivity for SARS-CoV-2 neutralizing activity among PLWH who received inactivated vaccines. Previous studies showed that the seroconversion rate of PLWH after inoculation with the hepatitis B vaccine ranged from 34% to 88% [31]. Therefore, we assumed that 70% of vaccinated PLWH would be positive for SARS-CoV-2 neutralizing activity. Using an allocation ratio of 2:1, a total of 102 PLWH and 51 HIV-negative individuals were required to detect a minimum between-group difference of 20% (90% versus 70%) in the positivity rate of SARS-CoV-2 neutralizing activity (α = 0.05, β = 0.10).

### 2.6. Statistical Analysis

Chi-square tests were used to inspect the difference in background characteristics and adverse events related to SARS-CoV-2 vaccination between PLWH and HIV-negative individuals. Between-group differences in immunogenicity indicator levels (total antibody, neutralizing activity, S-IgG, and T-cell-specific immune response) were tested using Mann–Whitney tests. We log transformed the immunogenicity indicator levels using a base of 10 to normalize the data. Multivariable linear regression models were performed to test the between-group differences in these indicators, after controlling for all background characteristics with *p* < 0.05 in between-group comparisons. Adjusted coefficients (B) were obtained. Moreover, same comparisons were performed between different subgroups of PLWH and HIV-negative individuals. Similar analyses on seropositivity for these immunogenicity indicators were also performed. Among PLWH, linear regression models were used to inspect factors that were correlated with immunogenicity indicator levels. In addition, correlations between CD4+ T-cell count/viral load and years since diagnosis were investigated. SPSS version 26.0 was used in all analyses, with two-tailed *p* < 0.05 considered statistically significant.

### 2.7. Ethics Statement

Written informed consent was obtained from all participants before their study participation in accordance with the Declaration of Helsinki. The Institutional Review Boards of Changzhi Medical College (RT2021002) and Beijing Youan Hospital Research Ethics Committee (No. 2021-031) approved this study.

## 3. Results

### 3.1. Profiles of the Participants

A total of 519 and 316 PLWH in Beijing and Tianjin were approached. A total of 130 and 24 from each city, respectively, were screened as eligible, and 110 (84.6%) and 19 (79%), respectively, completed the study. During the same period, 61 vaccinated HIV-negative individuals were approached. Eight (13.1%) refused to participate mainly due to logistical reasons, and 53 (86.9%) completed the study procedures. Most PLWH had received HIV diagnoses over one year ago (86%), and most were on ART (97.7%). Over half of them had an undetectable viral load (58.1%). The median HIV viral load and CD4+ T-cell counts were 42 (IQR: 0, 117) and 630.5 (IQR: 499.5, 848.8), respectively (Table 1).

As compared to HIV-negative individuals, fewer PLWH were 50–59 years old (3.9% versus 17.0%, *p* = 0.01) and female (0.8% versus 24.5%, *p* < 0.001). More PLWH had chronic conditions (20.9% versus 0%, *p* < 0.001), received Sinovac CoronaVac (55.0% versus 30.2%, *p* < 0.001), and had received only the first dose (27.1% versus 3.8%, *p* < 0.001). No participants had received more than one type of vaccine. Among those who completed both doses, the time interval between the first and second dose was shorter among PLWH than among HIV-negative individuals (median: 21 versus 27 days, *p* < 0.001) (Table 1). These background characteristics were controlled when comparing immunogenicity indicator levels between PLWH and HIV-negative individuals.

### 3.2. SARS-CoV-2 Vaccination Adverse Events

Among the participants, 45.0% of PLWH and 54.7% of HIV-negative individuals reported experiencing specific local or systematic adverse events. After controlling for significant background characteristics (i.e., age group, gender, presence of chronic conditions other than HIV, type of vaccine, time interval between first and second dose, and time after receiving the second dose), there was no between-group difference in prevalence of adverse events (AOR: 0.77, 95% CI: 0.31, 1.95, *p* = 0.19). Most of the reported adverse events were very mild/mild (41–100% among PLWH and 62.2–100% among HIV-negative individuals). There was no between-group difference in the severity of these adverse events (*p* = 0.13–0.77) (Table 2).

### 3.3. Immunogenicity Indicator Levels

The seropositivity of neutralizing activity, the total antibody, and the S-IgG values were 71.3, 81.9 and 92.6%, respectively, among fully vaccinated PLWH. The prevalence of seropositivity of all four immunogenicity indicators was lower among fully vaccinated PLWH than among fully vaccinated HIV-negative individuals, with the exception of neutralizing activity. (Table 3 and Table S1).

When compared to HIV-negative individuals, PLWH had significantly lower levels of neutralizing activity (adjusted B: −0.18, *p* = 0.049), total antibody (adjusted B: −0.80, *p* < 0.001), S-IgG (adjusted B: −0.31, *p* = 0.002), and T-cell-specific immune response (adjusted B: −0.64, *p* = 0.002). Subgroup analyses showed that all subgroups of PLWH had significantly lower neutralizing activity than HIV-negative individuals, with the exception of the subgroup of PLWH with CD4+ T-cell counts ≥500 and with undetectable viral loads (adjusted B: −0.23, *p* = 0.16). In addition, PLWH had significantly lower levels of total antibody, S-IgG, and T-cell-specific immune response, regardless of CD4+ T-cell counts or levels of HIV viral suppression. Neutralizing activity levels among fully vaccinated PLWH were not lower than those among fully vaccinated HIV-negative individuals (adjusted B: −0.15, *p* = 0.13) (Table 4 and Table 5).

### 3.4. Factors Associated with Immunogenicity Indicator Levels among PLWH

A longer time since HIV diagnosis was associated with higher neutralizing activity and total antibody levels (2–5 years: adjusted B: 0.71 and 0.27; reference: ≤1 year). As compared to partially vaccinated participants, PLWH who had received their second dose 15–28 days previously had higher neutralizing activity levels (adjusted B: 0.35), while those who had received it 15–56 days previously had higher levels of total antibody (adjusted B: 1.03), S-IgG (adjusted B: 0.73 and 0.74), and T-cell-specific immune response (adjusted B: 0.89 and 0.99). Compared to PLWH with a time interval of <21 days between the first and second dose, those with an interval of 21–28 days and >28 days had higher neutralizing activity (adjusted B: 0.37 and 0.36), total antibody (adjusted B: 0.93 and 1.15), and S-IgG levels (adjusted B: 0.43 and 0.53) (Table 6 and Table S2). A longer time since HIV diagnosis was correlated with higher CD4+ T-cell counts (r = 0.19, *p* = 0.03). However, there was no significant correlation between HIV viral load and time since HIV diagnosis (r = −0.004, *p* = 0.97).

## 4. Discussion

Understanding the differences in immunoresponse between HIV-negative and HIV-positive individuals is essential in planning SARS-CoV-2 vaccination for PLWH. We found the levels of adverse events to be comparable between PLWH and HIV-negative individuals. Although there was no significant between-group difference in seropositivity or levels of neutralizing activity, fully vaccinated PLWH had lower seropositivity and lower levels of total antibody, S-IgG, and T-cell-specific immune responses than fully vaccinated HIV-negative individuals. Our findings fill the knowledge gap concerning immune responses to SARS-CoV-2 vaccines among PLWH. They contribute critical evidence for policymaking and vaccination-program planning for countries that mainly use inactivated SARS-CoV-2 vaccines.

Similar to studies on mRNA/adenovirus vector SARS-CoV-2 vaccines [20,21,22,23,24], there was no between-group difference in prevalence (*p* = 0.19) or severity (*p* = 0.13–0.77) of self-reported adverse events. Most of the reported adverse events were very mild/mild among PLWH (41–100%). Therefore, inactivated SARS-CoV-2 vaccines are safe for PLWH.

Four immunogenicity indicator levels were significantly lower among PLWH at 0–14 days after receiving the second dose. PLWH might take longer to develop humoral and cellular immune responses to inactivated SARS-CoV-2 vaccines. Previous case reports observed a prolonged course of antibody development among PLWH infected with SARS-CoV-2 [32]. The studied immunogenicity indicators peaked at 15–56 days after the second dose among PLWH, a finding in line with previous research conducted with HIV-negative individuals and PLWH who received other SARS-CoV-2 vaccines [20,21,22,23,24]. However, the peak levels of these indicators were lower among PLWH, especially for total antibody and S-IgG. A faster decline in immune responses was also observed among PLWH. All four immunogenicity indicator levels declined >56 days after receiving the second dose among PLWH, while these indicators remained stable among HIV-negative individuals even 84 days after the second dose. This study observed significantly lower total antibody and S-IgG levels among PLWH >56 days after the second dose. B-cell dysfunction caused by HIV gp120 binding directly to primary B cells, and impaired cellular immunity caused by CD4+ T-cell depletion among PLWH, might explain the slower development, lower peak levels, and faster decline of both humoral and cellular immune responses to SARS-CoV-2 vaccines that we observed [33,34]. Such findings indicate that PLWH might need a booster dose after the primary series and might need it earlier than HIV-negative individuals. Future studies with large sample sizes are needed to investigate long-term changes in these immunogenicity indicators among PLWH.

Neutralizing antibody plays an important role in SARS-CoV-2 clearance and is a key indicator of protection after vaccination [35]. We found that seropositivity and levels of neutralizing activity were similar among fully vaccinated PLWH and HIV-negative individuals. This implies that both groups obtained good protection against SARS-CoV-2 after the vaccination and PLWH should complete both doses of vaccination as required. Subgroup analysis showed that PLWH with higher CD4+ T-cell counts and undetectable viral loads did not have significantly lower neutralizing activity levels than HIV-negative individuals, a finding in line with previous studies examining mRNA and/or adenovirus vector SARS-CoV-2 vaccines [20,21,22,23,24]. However, PLWH with lower CD4+ T-cell counts (<500) and/or detectable viral loads had lower neutralizing activity levels. Such findings add to knowledge regarding immune responses to SARS-CoV-2 vaccines among PLWH with more severe immunodeficiency. PLWH with more severe immunodeficiency should be encouraged to receive SARS-CoV-2 vaccines. In contrast to findings regarding other types of vaccines, our study observed significantly lower total antibody, S-IgG, and T-cell-specific immune response levels among PLWH compared to HIV-negative individuals. These differences could not be fully explained as a result of the larger proportion of PLWH with low CD4+ T-cell counts or detectable HIV viral loads in this study. These indicators were lower among PLWH regardless of their CD4+ T-cell counts or HIV viral loads. Future studies should compare PLWH’s immunogenicity in response to different types of SARS-CoV-2 vaccines in order to determine the optimal choice of vaccine for PLWH.

Compared to newly diagnosed PLWH, those who had been diagnosed 2–5 years prior to the commencement of the study had higher neutralizing activity and total antibody levels. Since increased length of time since HIV diagnosis was correlated with higher CD4+ T-cell counts, it is possible that these PLWH had better-functioning immune systems and hence higher neutralizing activity and antibody titers. This finding also highlights the need to further increase HIV testing coverage among key populations to improve early identification of HIV infection and direct these individuals toward treatment and care, hence improving the effectiveness of SARS-CoV-2 vaccination for PLWH. Moreover, our results also suggest that PLWH who had a longer interval between the first and second dose (21–28 days or >28 days) had significantly higher neutralizing activity, total antibody, and S-IgG levels compared to those with a shorter interval between doses. Existing guidelines regarding SARS-CoV-2 vaccination for PLWH do not mention the optimal vaccination interval. Our findings suggest that future SARS-CoV-2 vaccination programs for PLWH should consider a longer interval between doses. However, more research is needed to confirm the optimal interval between doses for PLWH.

This study has several strengths. First, we measured neutralizing activity using the gold-standard neutralization assay. Second, all participants underwent humoral and cellular immune response analyses in this study. Third, this study included a diverse sample of PLWH with different CD4+ T-cell levels and HIV viral loads, helping to fill the knowledge gap regarding the immunogenicity of SARS-CoV-2 vaccines among PLWH with impaired immune system functioning and poorer control of HIV. Fourth, the impact of between-group differences in background characteristics on immunogenicity may have been reduced in this study, since background characteristics were controlled during the comparison. Furthermore, this is also one the first studies to assess relationships between characteristics of PLWH and their immunogenicity in response to SARS-CoV-2 vaccines.

This study also has some limitations. Gender imbalance was one such factor. Gender is a biological variable that affects the functioning of the immune system. Generally, adult females mount stronger innate and adaptive immune responses than males, as noted in [36]; however, this previous study did not show any difference in immunogenicity in response to SARS-CoV-2 vaccination between males and females. Second, this was a cross-sectional study. Possible changes in immunogenicity indicator levels over time were unclear. Such a study design is also unable to establish causal relationships. Third, we did not match the sample of HIV-negative individuals according to the PLWH’s characteristics. There are significant between-group differences in socio-demographics, presence of other chronic conditions, and vaccination characteristics. However, we controlled these characteristics when comparing the between-group differences in immunogenicity. In addition, the presence and severity of adverse events were self-reported by participants and might have been subject to recall bias. This means we were also unable to compare the safety data with other studies that used clinician assessments.

## 5. Conclusions

Inactivated SARS-CoV-2 vaccines are safe for PLWH. Although there was no difference in the level of neutralizing activity, fully vaccinated PLWH had significantly lower total antibody, S-IgG, and T-cell-specific immune response levels than fully vaccinated HIV-negative individuals. The immunogenicity indicator levels peaked 15–56 days after PLWH receiving the second dose. A longer time since diagnosis and a longer interval between the first and second dose were correlated with better immune responses among PLWH. Future studies should compare PLWH’s immunogenicity in response to different types of vaccines, assess immune responses over a longer term, and investigate the optimal interval between doses for this population.

## Figures and Tables

**Table 1:** Background characteristics of HIV-negative individuals and people living with HIV (PLWH) who had received at least one dose of SARS-CoV-2 vaccine.

	People Living with HIV (n = 129)	HIV-Negative Individuals (n = 53)	*p* Values
**Socio-Demographics**			
Age (years), n (%)			
18–29	39 (30.2)	14 (26.4)	
30–39	65 (50.4)	19 (35.8)	
40–49	20 (15.5)	11 (20.8)	
50–59	5 (3.9)	9 (17.0)	0.01
Median (IQR), range	34 (28, 38) (20–58)	34 (29, 47) (22–56)	0.15
Gender, n (%)			
Male	128 (99.2)	40 (75.5)	
Female	1 (0.8)	13 (24.5)	<0.001
Presence of chronic conditions other than HIV/AIDS			
No	102 (79.1)	53 (100.0)	
Yes	27 (20.9)	0 (0.0)	<0.001
**Characteristics related to HIV infection**			
Time since HIV diagnosis (years)			
≤1	18 (14.0)	N.A	N.A.
2–5	55 (42.6)	N.A	N.A.
6–10	35 (27.1)	N.A	N.A.
>10	21 (16.3)	N.A	N.A.
Viral load (cp/mL), n (%)			
Undetectable (≤60)	75 (58.1)	N.A.	N.A.
61–200	33 (25.6)	N.A.	N.A.
>200	21 (16.3)	N.A.	N.A.
Median (IQR), range	41 (0, 117) (0, 50397)	N.A.	N.A.
CD4+ T-cell count (cells/μL)			
<500	32 (24.8)	N.A.	N.A.
500–1000	81 (62.8)	N.A.	N.A.
>1000	16 (12.4)	N.A.	N.A.
Median (IQR), range	630.5 (499.5, 848.8) (78, 2650.35)	N.A.	N.A.
ART regimens			
TDF + 3TC + EFV	60 (52.7)	N.A.	N.A.
TDF + 3TC + LPV/r	5 (3.9)	N.A.	N.A.
AZT + 3TC + LPV/r	3 (2.3)	N.A.	N.A.
AZT + 3TC + NVP	2 (1.6)	N.A.	N.A.
AZT + 3TC + EFV	8 (6.2)	N.A.	N.A.
Others	40 (31.0)	N.A.	N.A.
Not on ART	3 (2.3)	N.A.	N.A.
**Information related to SARS-CoV-2 vaccination**			
SARS-CoV-2 vaccination status			
Partially vaccinated	35 (27.1)	2 (3.8)	
0–14 days since fully vaccinated	15 (11.6)	8 (15.1)	
15–28 days since fully vaccinated	38 (29.5)	13 (25.5)	
29–56 days since fully vaccinated	26 (20.2)	21 (39.6)	
57–84 days since fully vaccinated	12 (9.3)	3 (5.7)	
>84 days since fully vaccinated	3 (2.3)	8 (15.1)	<0.001
Type of SARS-CoV-2 vaccine			
Sinopharm	58 (45.0)	37 (69.8)	
Sinovac CoronaVac	71 (55.0)	16 (30.2)	<0.001
Time interval between the first and second dose (among those who were fully vaccinated)	n = 94	n = 51	
<21 days	20 (21.3)	3 (5.7)	
21–28 days	58 (61.7)	40 (75.5)	
>28 days	16 (17.0)	10 (18.9)	0.043
Median (IQR), range	21 (21, 27) (14–59)	27 (21, 28) (14–83)	0.002

N.A.: not applicable.

**Table 2:** Comparing self-reported local and systematic adverse events related to SARS-CoV-2 vaccination among People living with HIV (PLWH) and HIV-negative individuals.

	People Living with HIV (n = 129)	HIV-Negative Individuals (n = 53)	*p* Values
	n (%)	n (%)	
**Local Adverse Events**			
Pain			
None	87 (67.4)	31 (58.5)	
Very mild	15 (11.6)	4 (7.5)	
Mild	16 (12.4)	11 (20.8)	
Moderate	11 (8.5)	7 (13.2)	
Severe	0 (0.0)	0 (0.0)	0.30
Any of above	42 (32.6)	22 (41.5)	0.25
Redness, itch, swelling, induration, and/or skin rash			
None	124 (96.1)	50 (94.3)	
Very mild	0 (0.0)	1 (1.9)	
Mild	2 (1.6)	2 (3.8)	
Moderate	3 (2.3)	0 (0.0)	
Severe	0 (0.0)	0 (0.0)	0.21
Any of above	5 (3.9)	3 (5.7)	0.59
**Systematic adverse events**			
Fatigue, malaise, headache, dizziness, and/or lethargy			
None	107 (82.9)	43 (81.1)	
Very mild	5 (3.9)	3 (5.7)	
Mild	11 (8.5)	4 (7.5)	
Moderate	5 (3.9)	2 (3.8)	
Severe	1 (0.8)	1 (1.9)	0.94
Any of above	22 (17.1)	10 (18.9)	0.77
Joint pain and/or muscle ache			
None	119 (92.2)	45 (84.9)	
Very mild	4 (3.1)	1 (1.9)	
Mild	3 (2.3)	4 (7.5)	
Moderate	3 (2.3)	3 (5.7)	
Severe	0 (0.0)	0 (0.0)	0.23
Any of above	10 (7.8)	8 (15.1)	0.13
Fever			
None	122 (94.6)	52 (98.1)	
Very mild	2 (1.6)	0 (0.0)	
Mild	4 (3.1)	1 (1.9)	
Moderate	1 (0.8)	0 (0.0)	
Severe	0 (0.0)	0 (0.0)	0.69
Any of above	7 (5.4)	1 (1.9)	0.27
Nausea, vomit, and/or diarrhea			
None	129 (100.0)	52 (98.1)	
Very mild	0 (0.0)	0 (0.0)	
Mild	0 (0.0)	1 (1.9)	
Moderate	0 (0.0)	0 (0.0)	
Severe	0 (0.0)	0 (0.0)	0.12
Any of above	0 (0.0)	1 (1.9)	0.29
Other systematic side-effects			
None	127 (98.4)	53 (100.0)	
Very mild	2 (1.6)	0 (0.0)	
Mild	0 (0.0)	0 (0.0)	
Moderate	0 (0.0)	0 (0.0)	
Severe	0 (0.0)	0 (0.0)	0.36
Any of above	2 (1.6)	0 (0.0)	0.50
Any local and/or systematic adverse events	58 (45.0)	29 (54.7)	0.23

**Table 3:** Seropositivity of SARS-CoV-2 neutralizing activity, total antibody, S-IgG, and T-cell-specific immune response among HIV-negative individuals and people living with HIV (PLWH) who had received at least one dose of SARS-CoV-2 vaccine.

	Neutralizing Activity			Total Antibody			S-IgG			T-Cell-Specific Immune Response		
	PLWH	HIV-Negative	*p*	PLWH	HIV-Negative	*p*	PLWH	HIV-Negative	*p*	PLWH	HIV-Negative	*p*
	% (n/N)	% (n/N)		% (n/N)	% (n/N)		% (n/N)	% (n/N)		% (n/N)	% (n/N)	
Partially vaccinated	5.7 (2/35)	50.0 (1/2)	0.03	31.4 (11/35)	50.0 (1/2)	0.55	31.4 (11/35)	100 (2/2)	0.12	20.0 (7/35)	50.0 (1/2)	0.32
0–14 days since fully vaccinated	33.3 (5/15)	75 (6/8)	0.09	46.7 (7/15)	100 (8/8)	0.01	66.7 (10/15)	100 (8/8)	0.09	33.3 (5/15)	100 (8/8)	0.01
15–28 days since fully vaccinated	81.6 (31/38)	90.9 (10/11)	0.66	94.7 (36/38)	100 (11/11)	0.60	100 (38/38)	100 (11/11)	N.A.	65.8 (25/38)	81.8 (9/11)	0.27
29–56 days since fully vaccinated	84.6 (22/26)	85.7 (18/21)	0.62	88.5 (23/26)	95.2 (20/21)	0.41	96.2 (25/26)	100 (21/21)	0.55	53.8 (14/26)	81.0 (17.21)	0.049
57–84 days since fully vaccinated	58.3 (7/12)	100 (3/3)	0.26	66.7 (8/12)	100 (3/3)	0.36	91.7 (11/12)	100 (3/3)	0.80	16.7 (2/12)	100 (3/3)	0.02
>84 days since fully vaccinated	66.7 (2/3)	62.5 (5/8)	0.72	100 (3/3)	100 (8/8)	N.A.	100 (3/3)	100 (8/8)	N.A.	33.3 (1/3)	50.0 (4/8)	0.62
Among all participants	53.5 (69/129)	81.1 (43/53)	<0.001	68.2 (88/129)	96.2 (51/53)	<0.001	76.0 (98/129)	100 (53/53)	<0.001	41.9 (54/129)	79.2 (42/53)	<0.001
Among participants who were fully vaccinated	71.3 (67/94)	82.4 (42/51)	0.14	81.9 (77/94)	98.0 (50/51)	0.005	96.2 (87/94)	100 (51/51)	0.04	50.0 (47/94)	80.4 (41/51)	<0.001

*p* values were obtained by using Chi-square tests or Fisher exact tests. N.A.: not applicable.

**Table 4:** Levels of SARS-CoV-2 neutralizing activity, total antibody, S-IgG, and T-cell-specific immune response among HIV-negative individuals and people living with HIV (PLWH) who had received at least one dose of SARS-CoV-2 vaccine.

	Neutralizing Activity			Total Antibody			S-IgG			T-Cell-Specific Immune Response		
	PLWH	HIV- Negative	*p*	PLWH	HIV-Negative	*p*	PLWH	HIV-Negative	*p*	PLWH	HIV- Negative	*p*
	GMT (95%CI)	GMT (95%CI)		Median (IQR)	Median (IQR)		Median (IQR)	Median (IQR)		Median (IQR)	Median (IQR)	
Partially vaccinated	4.6 (4.0, 9.8)	5.6 (N.A.)	0.43	0.2 (0.02, 1.1)	2.1 (N.A.)	0.20	0.6 (0.3, 1.5)	3.99 (N.A.)	0.03	6.4 (0.2, 26.7)	36.2 (N.A.)	0.16
0–14 days since fully vaccinated	8.5 (4.0, 64.6)	31.6 (4.0, 257.0)	0.03	0.8 (0.03, 16.8)	104.8 (7.4, 279.5)	0.01	3.1 (1.1, 16.2)	11.9 (5.1, 55.5)	0.04	5.3 (0.1, 88.8)	413.6 (91.8, 575.5)	0.001
15–28 days since fully vaccinated	24.0 (4.0, 380.2)	23.4 (4.0, 64.0)	0.97	28.9 (7.4, 83.2)	40.3 (28.5, 71.6)	0.24	9.0 (4.6, 16.0)	13.9 (10.1, 32.0)	0.13	56.08 (19.6, 118.7)	91.54 (31.1, 227.4)	0.29
29–56 days since fully vaccinated	14.1 (4.0, 64.6)	20.9 (4.0, 190.5)	0.24	11.8 (5.7, 27.3)	42.7 (8.4, 74.9)	0.04	7.2 (4.5, 12.2)	9.6 (7.2, 21.9)	0.03	37.2 (6.4, 121.1)	63.6 (35.4, 182.1)	0.13
57–84 days since fully vaccinated	11.0 (4.0, 95.5)	26.3 (12.0, 64.0)	0.18	6.2 (0.5, 11.7)	33.4 (N.A.)	0.04	3.4 (1.4, 5.7)	10.5 (N.A.)	0.03	3.6 (0.1, 17.1)	205.5 (N.A.)	0.08
>84 days since fully vaccinated	6.3 (4.0, 8.0)	11.1 (4.0, 48.0)	0.50	3.0 (1.3, N.A.)	9.3 (4.0, 62.8)	0.15	3.8 (1.2, N.A.)	4.3 (2.9, 5.4)	0.31	18.3 (0.8, N.A.)	35.6 (13.5, 56.2)	0.41
Among all participants	11.0 (4.0, 95.5)	20.0 (4.0, 190.5)	0.001	5.6 (0.4, 25.2)	32.6 (8.4, 72.3)	<0.001	4.3 (1.2, 10.0)	9.6 (5.4, 18.9)	<0.001	18.7 (2.4, 77.9)	63.6 (36.0, 226.4)	<0.001
Among participants who were fully vaccinated	15.1 (4.0, 128.8)	20.9 (4.0, 190.5)	0.09	10.3 (2.3, 38.8)	33.4 (10.1, 73.0)	<0.001	6.8 (3.3, 12.1)	10.1 (6.5, 19.4)	0.007	30.6 (5.2, 103.2)	68.4 (36.1, 227.4)	0.001

*p* values were obtained by using Mann–Whitney tests. N.A.: not applicable.

**Table 5:** Comparing immunogenicity indicator levels between different subgroups of people living with HIV (PLWH) and HIV-negative individuals.

	Neutralizing Activity		Total Antibody		S-IgG		T-Cell-Specific Immune Response	
	Adjusted B (95%CI)	*p* Values	Adjusted B (95%CI)	*p* Values	Adjusted B (95%CI)	*p* Values	Adjusted B (95%CI)	*p* Values
Reference 1: HIV-negative individuals (*n* = 53)	Ref	Ref	Ref	Ref	Ref	Ref	Ref	Ref
PLWH (n = 129)	−0.18 (−0.36, −0.001)	0.049	−0.80 (−1.15, −0.46)	<0.001	−0.31 (−0.51, −0.12)	0.002	−0.64 (−1.05, −0.23)	0.002
PLWH with CD4^+^ T-cell counts < 500 and detectable viral load (*n* = 13)	−0.69 (−1.02, −0.36)	<0.001	−2.51 (−3.01, −2.01)	<0.001	−1.16 (−1.44, −0.88)	<0.001	−1.35 (−1.94, −0.76)	<0.001
PLWH with CD4^+^ T-cell counts < 500 and undetectable viral load (*n* = 19)	−0.21 (−0.40, −0.01)	0.04	−1.05 (−1.57, −0.54)	<0.001	−0.38 (−0.67, −0.09)	0.01	−0.91 (−1.39, −0.42)	<0.001
PLWH with CD4+ T-cell counts ≥ 500 and detectable viral load (*n* = 41)	−0.32 (−0.57, −0.08)	0.01	−1.32 (−1.81, −0.82)	<0.001	−0.72 (−1.02, −0.42)	<0.001	−0.98 (−1.41, −0.55)	<0.001
PLWH with CD4+ T-cell counts ≥ 500 and undetectable viral load (*n* = 56)	−0.23 (−0.57, 0.10)	0.16	−0.72 (−1.05, −0.40)	<0.001	−0.32 (−0.51, −0.13)	0.001	−0.46 (−0.84, −0.08)	0.02
Reference 2: Fully vaccinated HIV-negative individuals (*n* = 51)	Ref	Ref	Ref	Ref	Ref	Ref	Ref	Ref
Fully vaccinated PLWH (*n* = 94)	−0.15 (−0.35, 0.04)	0.13	−0.68 (−1.03, −0.33)	<0.001	−0.27 (−0.48, −0.07)	0.01	−0.61 (−1.00, −0.22)	0.002

Ref: reference group. Adjusted B: adjusted correlation coefficients, adjusted for background characteristics with significant between-group differences in Table 1 (age group, gender, presence of chronic conditions other than HIV, types of SARS-CoV-2 vaccine, time interval between first and second dose, and SARS-CoV-2 vaccination status).

**Table 6:** Factors associated with SARS-CoV-2 total antibody, neutralizing antibody, S-IgG, and T-cell-specific immune response levels among people living with HIV (PLWH) (*n* = 129).

	Total Antibody		Neutralizing Activity		S-IgG		T-Cell-Specific Immune Response	
	Adjusted B (95%CI)	*p* Values	Adjusted B (95%CI)	*p* Values	Adjusted B (95%CI)	*p* Values	Adjusted B (95%CI)	*p* Values
Characteristics related to HIV infection								
Years since HIV diagnosis (years)								
≤1	Ref	Ref	Ref	Ref				
2–5	0.71 (0.23, 1.19)	0.004	0.27 (0.05, 0.48)	0.02				
6–10	0.49 (−0.03, 1.00)	0.07	0.23 (−0.01, 0.46)	0.06				
>10	0.44 (−0.13, 1.05)	0.13	0.15 (−0.11, 0.20)	0.24	---	---	---	---
Viral load (cp/mL)								
Undetectable	Ref	Ref			Ref	Ref	Ref	Ref
61–200	−0.24 (−0.61, 0.14)	0.21			−0.19 (−0.40, 0.03)	0.08	−0.01 (−0.44, 0.42)	0.97
>200	−0.24 (−0.69, 0.22)	0.31	---		−0.24 (−0.50, 0.03)	0.08	−0.27 (−0.78, 0.25)	0.30
CD4+ T-cell count (cells/μL)								
<500							Ref	Ref
500–1000							0.47 (0.05, 0.89)	0.03
>1000	---	---	---	---	---	---	0.40 (−0.22, 1.01)	0.20
On ART								
No								
Yes	---	---	---	---	---	---	---	---
SARS-CoV-2 vaccination								
SARS-CoV-2 vaccination status								
Partially vaccinated	Ref	Ref	Ref	Ref	Ref	Ref	Ref	Ref
0–14 days since fully vaccinated	0.03(−0.36, 0.63)	0.92	0.06 (−0.21, 0.32)	0.68	0.21 (−0.13, 0.54)	0.022	0.16 (−0.46, 0.78)	0.60
15–28 days since fully vaccinated	1.03 (0.43, 1.57)	0.001	0.35 (0.08, 0.63)	0.01	0.74 (0.39, 1.08)	<0.001	0.99 (0.50, 1.47)	<0.001
29–56 days since fully vaccinated	1.03 (0.47, 1.59)	<0.001	0.21 (−0.09, 0.46)	0.10	0.73 (0.41, 1.05)	<0.001	0.89 (0.37, 1.40)	0.001
57–84 days since fully vaccinated	0.85 (0.15, 1.55)	0.01	0.19 (−0.11, 0.49)	0.21	0.47 (0.09, 0.84)	0.02	−0.10 (−0.76, 0.57)	0.77
>84 days since fully vaccinated	0.29 (−0.83, 1.41)	0.58	−0.25 (−0.77, 0.26)	0.34	0.27 (−0.37, 0.93)	0.41	0.20 (−1.01, 1.41)	0.74
Type of SARS-CoV-2 vaccine								
Sinopharm	Ref	Ref	Ref	Ref	Ref	Ref		
Sinovac CoronaVac	0.26 (−0.07, 0.59)	0.12	0.05 (−0.10, 0.20)	0.50	0.07 (−0.11, 0.25)	0.45	---	---
Time interval (days) between the first and second dose								
<21 days	Ref	Ref	Ref	Ref	Ref	Ref		
21–28 days	0.93 (0.43, 1.43)	<0.001	0.37 (0.15, 0.59)	0.001	0.43 (0.14, 0.71)	0.003		
>28 days	1.15 (0.53, 1.77)	<0.001	0.36 (0.09, 0.63)	0.009	0.53 (0.18, 0.88)	0.003		
Not applicable (partially vaccinated)	N.A.	N.A.	N.A.	N.A.	N.A.	N.A.	---	---

Ref: reference group. Adjusted B: adjusted coefficients obtained from multivariate linear regression models using all significant variables as candidates. --- indicates *p* > 0.05 in univariate analysis and exclusion from multivariate analysis.

## Data Availability

The individual participant data used in this analysis are available upon request. Requests should be directed to the corresponding author and requesters must sign a data access and confidentiality agreement.

## Supplementary Materials

The following are available online at https://www.mdpi.com/article/10.3390/v14020277/s1. Table S1: Comparing seropositivity of immunogenicity indicators between different subgroups of PLWHA and HIV-negative individuals. Table S2: Unadjusted correlation coefficients of factors associated with SARS-CoV-2 total antibody, neutralizing activity, S-IgG, and T-cell-specific immune response levels among people living with HIV (PLWH) (n = 129).
