# Supplementary material for: Comparing Immune Responses to Inactivated Vaccines against SARS-CoV-2 between People Living with HIV and HIV-Negative Individuals: A Cross-Sectional Study in China
Source: Viruses. 2022 Jan 28;14(2):277. doi: 10.3390/v14020277 (PMC8875274; doi:10.3390/v14020277)
Supplement: Supplementary file 1 [file viruses-14-00277-s001.zip › viruses-1552498-supplementary.pdf]

Table S1. Comparing sero-positivity of immunogenicity indicators between different subgroups of PLWHA and HIV-negative individuals

	SARS-Cov-2 total antibody			SARS-Cov-2 neutralizing activity			S IgG			T cell specific immune response		
	%	AOR (95%CI)	P values	%	AOR (95%CI)	P values	%	AOR (95%CI)	P values	%	AOR (95%CI)	P values
Reference: Fully vaccinated HIV-negative individuals (n=51)	98.0	Ref	Ref	82.4	Ref	Ref	100	Ref	Ref	80.4	Ref	Ref
Fully vaccinated PLWHA (n=94)	81.9	0.12 (0.01, 1.15)	.07	71.3	0.65 (0.20, 2.10)	.48	92.6	N.A.	N.A.	50.0	0.12 (0.04, 0.38)	<.001
Fully vaccinated PLWHA with CD4+ T cell counts<500 and detectable viral load (n=7)	28.6	N.A.	N.A.	28.6	0.03 (0.001, 0.95)	.048	57.1	N.A.	N.A.	14.3	N.A.	N.A.
Fully vaccinated PLWHA with CD4+ T cell counts<500 and undetectable viral load (n=16)	81.3	N.A.	N.A.	56.3	0.26 (0.03, 1.24)	.24	100	N.A.	N.A.	43.8	0.30 (0.002, 0.54)	.02
Fully vaccinated PLWHA with CD4+ T cell counts≥500 and detectable viral load (n=28)	78.6	0.04 (0.001, 1.27)	.07	75.0	0.82 (0.12, 5.49)	.83	85.7	N.A.	N.A.	39.3	0.03 (0.01, 0.25)	.001
Fully vaccinated PLWHA with CD4+ T cell counts≥500 and undetectable viral load (n=43)	93.0	0.52 (0.01, 46.30)	.78	81.4	1.03 (0.20, 5.41)	.97	100	N.A.	N.A.	65.1	0.17 (0.03, 0.86)	.03

AOR: adjusted odds ratios, odds ratios adjusted for background characteristics with significant between-group difference in Table 1 (age group, gender, presence of chronic conditions other than HIV, vaccine type, time interval between first and second dose, and time since the completion of the second dose)

Table S2. Unadjusted correlation coefficients of factors associated with SARS-CoV-2 total antibody, neutralizing activity, S-IgG, and T cell specific immune response levels among people living with HIV (PLWH) (n=129).

	**Total antibody**		**Neutralizing activity**		**S-IgG**		**T cell specific immune response**	
	**Unadjusted B (95%CI)**	**P values**	**Unadjusted B (95%CI)**	**P values**	**Unadjusted B (95%CI)**	**P values**	**Unadjusted B (95%CI)**	**P values**
**Socio-demographics**								
Age (years)								
18-29	Ref	Ref	Ref	Ref	Ref	Ref	Ref	Ref
30-39	-0.06 (-0.57, 0.45)	.81	0.06 (-0.14, 0.25)	.58	0.03 (-0.26, 0.32)	.83	-0.07 (-0.51, 0.38)	.77
40-49	0.17 (-0.52, 0.86)	.63	0.08 (-0.18, 0.35)	.54	-0.09 (-0.48, 0.31)	.67	-0.08 (-0.69, 0.53)	.80
50-59	-0.32 (-1.51, 0.87)	.60	-0.03 (-0.49, 0.43)	.91	0.03 (-0.66, 0.71)	.94	-0.56 (-1.62, 0.49)	.29
Gender								
Male	Ref	Ref	Ref	Ref	Ref	Ref	Ref	Ref
Female	1.47 (-1.02, 3.97)	.24	0.77 (-0.18, 1.73)	.11	0.63 (-0.81, 2.06)	.39	1.14 (-1.07, 3.34)	.31
Presence of chronic conditions other than HIV/ADIS								
No	Ref	Ref	Ref	Ref	Ref	Ref	Ref	Ref
Yes	0.12 (-0.42, 0.66)	.66	-0.07 (-0.28, 0.14)	.52	-0.01 (-0.30, 0.32)	.95	-0.20 (-0.68, 0.28)	.41
**Characteristics related to HIV infection**								
Years since HIV diagnosis (years)								
≤1	Ref	Ref	Ref	Ref	Ref	Ref	Ref	Ref
2-5	0.55 (-0.12, 1.22)	.11	0.21 (-0.05, 0.46)	.12	0.21 (-0.18, 0.60)	.28	0.46 (-0.14, 1.06)	.13
6-10	0.82 (0.10, 1.53)*	.03	0.33 (0.05, 0.60)*	.02	0.33 (-0.09, 0.74)	.12	0.56 (-0.08, 1.19)†	.09
>10	0.59 (-0.20, 1.38)	.14	0.22 (-0.08, 0.53)	.15	0.23 (-0.23, 0.69)	.32	0.55 (-0.15, 1.26)	.12
Viral load (cp/mL)								
Undetectable	Ref	Ref	Ref	Ref	Ref	Ref	Ref	Ref
61-200	-0.39 (-0.89, 0.10)	.12	-0.17 (-0.36, 0.03)†	.099	-0.33 (-0.61, -0.05)*	.02	-0.15 (-0.61, 0.30)	.50
>200	-1.10)	<.001	-0.23	.051	-0.68	<.001	-0.60	.03

	(-1.69, -0.51)		(-0.47, 0.001)†		(-1.01, -0.34)		(-1.14, -0.06)*	
CD4+ T cell count (cells/μL)								
<500	Ref	Ref	Ref	Ref	Ref	Ref	Ref	Ref
500-1,000	0.41 (-0.10, 0.93)	.11	0.13 (-0.07, 0.33)	.20	0.16 (-0.14, 0.45)	.31	0.59 (0.14, 1.04)*	.01
>1,000	0.61 (-0.15, 1.36)	.11	0.14 (-0.15, 0.44)	.34	0.24 (-0.20, 0.68)	.28	0.48 (-0.18, 1.14)	.16
On ART								
No	Ref	Ref	Ref	Ref	Ref	Ref	Ref	Ref
Yes	0.47 (-0.99, 1.93)	.52	0.12 (-0.44, 0.68)	.67	0.13 (-0.71, 0.97)	.76	0.34 (-0.95, 1.62)	.61
**SARS-CoV-2 vaccination**								
SARS-CoV-2 vaccination status								
Partially vaccinated	Ref	Ref	Ref	Ref	Ref	Ref	Ref	Ref
0-14 days after fully vaccinated	0.68 (0.11, 1.25)*	.02	0.27 (0.02, 0.51)*	.03	0.49 (0.17, 0.80)**	.003	0.07 (-0.55, 0.69)	.82
15-28 days after fully vaccinated	2.11 (1.68, 2.55)	<.001	0.72 (0.54, 0.91)	<.001	1.26 (1.02, 1.50)	<.001	1.08 (0.61, 1.54)	<.001
29-56 days after fully vaccinated	1.79 (1.32, 2.27)	<.001	0.49 (0.28, 0.69)	<.001	1.08 (0.82, 1.35)	<.001	0.94 (0.42, 1.45)	<.001
57-84 days after fully vaccinated	1.36 (0.74, 1.97)	<.001	0.38 (0.12, 0.65)**	.01	0.75 (0.40, 1.09)	<.001	-0.07 (-0.74, 0.59)	.83
>84 days after fully vaccinated	1.31 (0.20, 2.41)*	.02	0.15 (-0.33, 0.62)	.55	0.69 (0.07, 1.31)*	.03	0.40 (-0.80, 1.60)	.51
Type of SARS-CoV-2 vaccine								
Sinopharm	Ref	Ref	Ref	Ref	Ref	Ref	Ref	Ref
Sinovac-CoronaVac	0.71 (0.28, 1.13)**	.001	0.23 (-0.07, 0.40)**	.006	0.31 (0.06, 0.55)*	.02	0.31 (-0.08, 0.69)	.12
Time interval (days) between the prime and second dose								
<21 days	Ref	Ref	Ref	Ref	Ref	Ref	Ref	Ref
21-28 days	1.22 (0.74, 1.69)***	<.001	0.42 (0.22, 0.64)	<.001	0.67 (0.41, 0.94)	<.001	0.52 (-0.02, 1.06)†	.06
>28 days	1.28 (0.67, 1.89)***	<.001	0.39 (0.12, 0.66)**	.005	0.70 (0.35, 1.04)	<.001	0.62 (-0.09, 1.32)†	.09
Not applicable (partially vaccinated)	-0.71 (-1.22, -0.19)**	.007	-0.19 (-0.42, 0.03)†	.09	-0.47 (-0.76, -0.18)**	.002	-0.28 (-0.87, 0.31)	.34
